# Trends and hotspots in running shoe research: a bibliometric study from 2005 to 2024

**DOI:** 10.3389/fspor.2025.1609141

**Published:** 2025-09-10

**Authors:** Xiaoge Xiao, Ao Lian, Zhiyu Li, Yifang Fan

**Affiliations:** ^1^ Foot Research Laboratory, School of Physical Education and Sport Science, Fujian Normal University, Fuzhou, China; ^2^ College of Foreign Studies, Jinan University, Guangzhou, China

**Keywords:** running shoe, running injuries, running performance, bibliometrics, visualization mapping

## Abstract

**Background:**

Running shoes can protect the feet, enhance performance and lower the injury risk during running. While extensive research has been investigated on footwear design and innovation in running, the scientific guideline underlying running shoe research remain inadequately explored and established.

**Purpose:**

The aims of this study was to conduct a bibliometric analysis of publications in running shoes for identifying research hotspots and future trends. The results from this study can provide valuable references for future studies and contribute to the scientific advancement of running shoe design.

**Method:**

Articles on running shoes were collected and screened from the Web of Science Core Collection database covering the years 2005–2024. After duplicate and irrelevant articles removed, CiteSpace, VOSviewer, and R-biblioshiny were used to perform visualized analyses of authors, titles, journals, countries, institutions, keywords, research directions, and cited references. Co-citation maps were created to provide a clear representation of research hotspots and knowledge structures.

**Result:**

A total of 1,576 articles on running shoes were identified across 394 journals spanned 69 countries and 3,599 institutions, with peak publication volume found in 2022. The United States generated the highest number of publications, followed by China and the United Kingdom. The University of Calgary produced the highest publication output. Gu YD was the top author to produce the most publications, while Lieberman DE was identified as the most influential scholar in the field. The *Medicine & Science in Sports & Exercise* have been the most prominent journals in this field. Trend keywords had centered on running injuries (e.g., “*barefoot*,” “*ground reaction force*,” and “*injuries*”) and performance (e.g., “*running economy*,” “*performance*,” and “*metabolic cost*”), which have been clustered into eight distinct labels.

**Conclusion:**

This is the first study to present bibliometric analysis on running shoes literature over the past 20 years, highlighting the key hotspots and future trends. Overall, the annual publications on running shoes has steadily increased. Current research have focused on the biomechanics and physiological indicators of runners whilst wearing running shoes to explore the associated injury risks and running performance, with particular emphasis on the impact of minimalist shoes.

## 1 Introduction

The significance of running shoes is increasingly investigated in modern sports science and public health (1). Since the late 20th century, researchers and manufacturers have focused on optimizing running shoe performance with various materials, structure, and biomechanics to enhance athletic performance and reduce injury risks (2, 3). Furthermore, as endurance events such as marathons and trail running have gained widespread popularity globally (4), the functional demands of running shoes have evolved continuously to give new technological advancements such as high rebound, lightweight, enhanced support, and smart monitoring (5–7). Researchers have explored improvements in running economy from both biomechanical and exercise physiology perspectives, as well as structural innovations driven by materials science and design engineering (8, 9). This multi-faceted, interdisciplinary research has advanced the running shoe research, further highlighting its significance across sports science, engineering technology, and industrial applications (10).

The running shoes has undergone several development stages. Early designs primarily focused on basic protection and comfort, and applied rigid materials to provide support and stability (11). However, these designs failed to effectively reduce the impact forces that experienced during activities (3, 12, 13). By the late 1980s, with advancements in biomechanics research, shoe designs began to shift towards greater emphasis on cushioning and comfort (14). Simultaneously, the design of midsole support gained greater attention (15), suggesting that appropriate support could effectively control excessive foot pronation or supination for alleviating the load on lower limb (16). In recent years, researchers have integrated sensor technology with big data analysis, utilized wearable devices to monitor key indicators in real-time during running and applied deep learning or artificial intelligence algorithms for prediction and analysis (17). These smart running shoes or insoles not only collect biomechanical parameters during the running but also optimize training strategies and rehabilitation programs (18). With the growing popularity of running, the running shoe market is increasingly developed with level-specific products such as comfort-specific shoes for novices and high-elasticity carbon-plated shoes targeting long-distance athletes, both emerging as hotspots for research and development (19). However, the multi-dimensional findings from biomechanics, materials science, and sports medicine are reported across different disciplines and journals, lacking a systematic and holistic review and synthesis. Traditional reviews or qualitative studies can summarize existing progress to some extent, but with the continuous increase of large and discrete literature in recent years, the challenge of quickly and accurately identifying core authors and research institutions, as well as tracking the historical evolution and cutting-edge trends in running shoe research, has become significant (20, 21). Bibliometric and scientific visualization analysis tools, such as CiteSpace and VOSviewer, can offer researchers an effective pathway to quantitatively analyzing target literature in databases and generating scientific visualizations (22). These tools not only objectively assess academic influence but also reveal the thematic focus and relational structure within knowledge networks, offering a valuable global reference for future research directions and technological advancements (23). To our best knowledge, however, there have been no bibliometric studies reported to running shoes.

To review the landscape of running shoe research, this study aimed to employ a comprehensive bibliometric analysis to systematically identify and evaluate high-quality literature on running shoe research published in Web of Science Core Collection database from 2005–2024. Specifically, we aimed to: 1. statistically analyze the distribution and collaboration networks of major international research institutions, scientific teams, and core authors; 2. identify frequently cited representative literature and key themes, and analyze the core issues and knowledge frameworks within running shoe research; and 3. explore and visualize the research hotspots and potential frontiers in this field, and discuss their implications for the subsequent design of running shoes for reducing running-related injuries and improving athletic performance. The findings from this study can offer a comprehensive overview of scientific output over time and further promote interdisciplinary collaboration in running shoe development and sports science research.

## 2 Methods

### 2.1 Data collection and search strategy

Although knowledge graph research typically requires larger datasets to obtain more comprehensive visual information, high-quality and accurate data can better reflect its value in mining and analysis. To identify the time frame for bibliometric research, the “high activity period”, that have shown a continuous increase in publication volume over the past few decades without significant declines was identified. the articles related to running shoes were retrieved using the algorithm of searching terms (“running shoe*” OR “running footwear*”) from the *Web of Science Core Collection* on March 31, 2025, resulting in a preliminary total of 2,169 articles.

**Figure 1:**
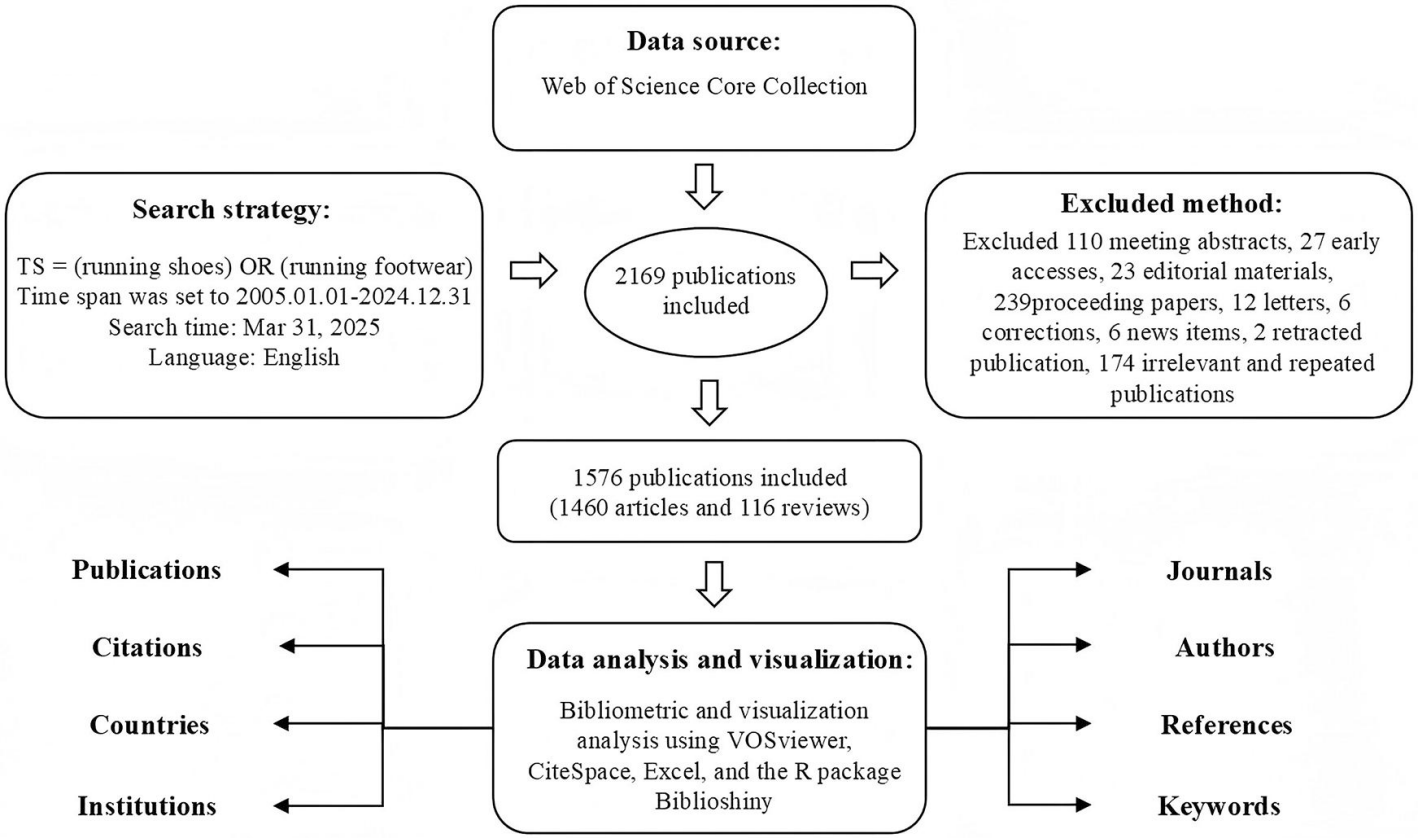
Study selection procedure.

The screening and data processing are illustrated in Figure 1, and the literature collection and screening criteria are listed as follows (24):

1. Two researchers independently conducted the screening and selection;
2. The publication date range was set from 2005 to 01-01 to 2024-12-31;
3. Only original articles (Article) or review articles (Review Article) were included;
4. The language was limited to English;
5. Duplicate literature was removed.

After screening and removing duplicate records, the data extract format and merged the data records to reduce data noise for this study was standardized such as author information and affiliations, merging of renamed journals, and processing and merging keywords (25) A total of 1,576 documents were identified, with 1,460 original articles (Article) and 116 review articles (Review Article) for subsequent analysis. The retrieved information included authors, titles, journals, countries, affiliations, keywords, research directions, and cited references.

### 2.2 Data analysis and visualization

A range of tools and techniques was employed for data analysis and visualization, including the R package Biblioshiny (https://bibliometric.com), VOSviewer 1.6.20, and CiteSpace 6.4.R2. These three major bibliometric software tools each focus on distinct areas of exploration. *VOSviewer* (ver. 1.6.18, Centre for Science and Technology Studies, Leiden University, Leiden, Netherlands) were employed to perform visualizing collaboration relationships among authors, co-occurrence analysis of keywords, co-authorship and co-citation analysis, as well as collaboration networks among institutions and countries (26). CiteSpace emphasizes keyword burst detection and timeline views of reference clusters, revealing influential literature and emerging research hotspots in different time periods, thereby illustrating the evolution of research (27). Biblioshiny was primarily used to extract basic information from all publications, included the number of publications, types of publications, number of publications by authors, number of publications by journals, and authors, as well as to visualize collaboration networks among countries (28).

## 3 Results

### 3.1 Analysis of publications and citations

**Figure 2:**
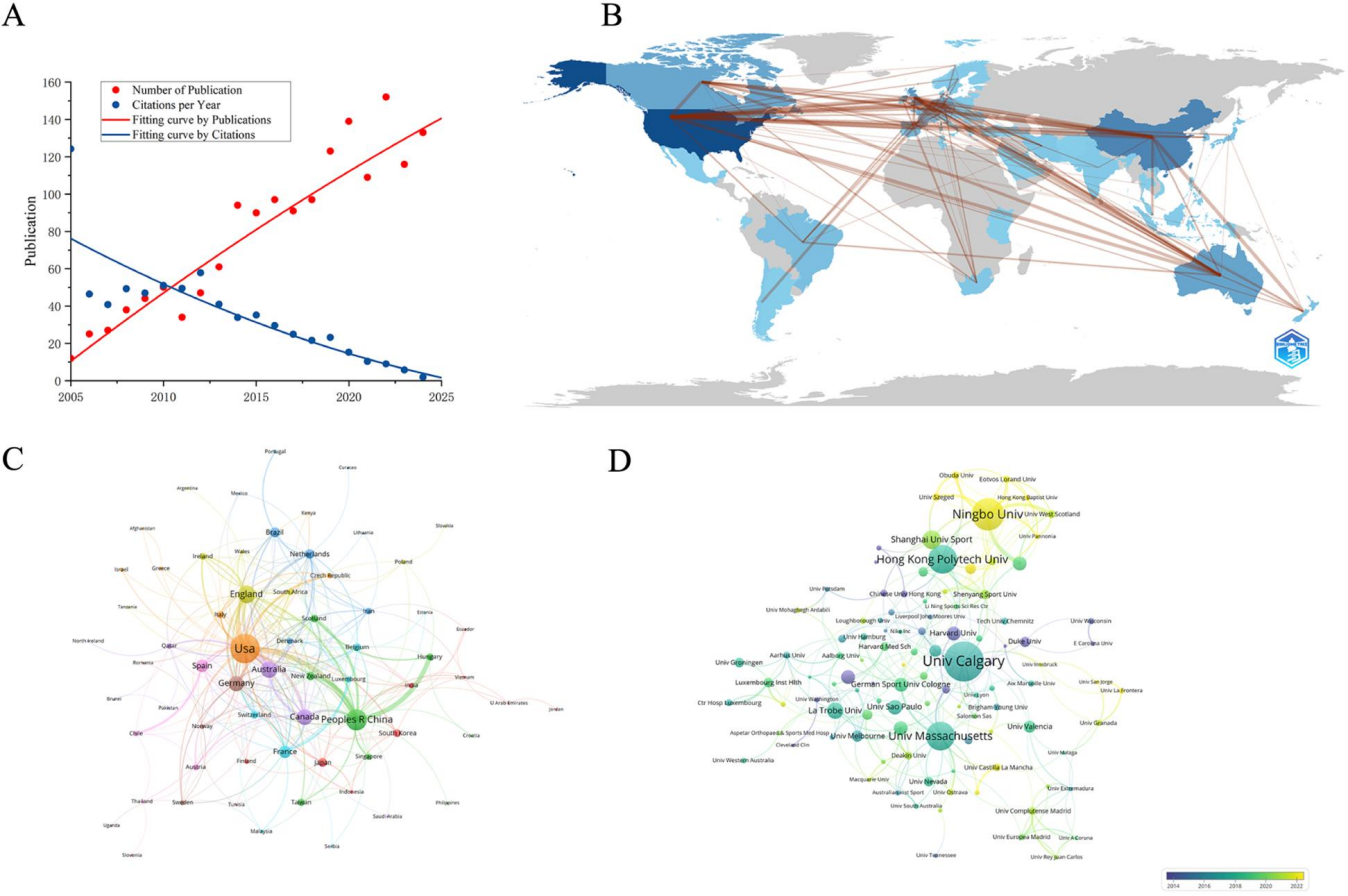
Visualization of national and institutional collaboration networks. **(A)** Trends in annual publications and citations from 2005–2024. **(B)** Country collaboration map: the lines connecting countries on the map represents collaborative links; the thickness of each line indicates the strength of collaboration between the respective countries. **(C)** The network map of countries: the size of the nodes represents the number of publications. **(D)** The co-authorship analysis of institutions: the size of the nodes represents the number of documents. The color represents the average year.

A total of 1,576 publications related to running shoes were retrieved across 394 journals by 4,892 authors from 3,599 institutions across 69 countries in the past 20 years. This includes 1,460 original articles and 116 review articles. The annual growth rate of publications was 13.5%, with an average citation count of 24.34 citations per article. The publication volume peaked in 2022, reaching 152 articles. In the early period (i.e., 2005–2010), the publication output remained relatively low and steady with an average of about 30 articles per year, until a significant increase began in 2011. Over the subsequent three years, the number of publications rose from 34–94. Another rapid growth phase commenced in 2018 with the publication output increased from 97–152 articles within four years (Figure 2A). These two phases of rapid growth would be associated with the introduction of the minimalist shoe and Nike Vaporfly 4% concepts.

### 3.2 Analysis of countries and funds

Among the published literature between 2005 and 2024, the United States had the highest publication volume, reaching 475 papers, which accounts for 30.1% of the total publication outputs (Table 1). China and United Kingdom ranked second and third with 236 publications (i.e., 15.0%) and 172 publications (i.e., 10.9%) respectively, followed by Australia and Canada for the fourth and fifth positions with 146 (9.3%) and 138 (8.8%). For citation counts, the United States also generated a total of 14,371 citations, which was significantly higher than other countries. Specifically, China, the United Kingdom, Australia, and Canada had 3,842, 2,865, 2,651, and 2,335 citations, respectively. Notably, the combined citation total of the latter four countries (11,693) was still lower than that of the United States, underscoring its dominant position in academic influence. To combine the centrality data (Table 1), the country collaboration map (Figure 2B) and the network map of countries (Figure 2C), it confirmed that the United States (0.41) and China (0.43) hold central positions within the collaboration network. Both countries generated in the number of publications and demonstrated frequent international collaboration to establish themselves as key hubs in this research domain. In terms of funding sources, National Natural Science Foundation of China ranked the first in publications with 85 publications and 1,556 citations. However, when considering average citation rates, United States Department of Health and Human Services led with 16 publications and 809 citations, followed closely by the National Institutes of Health with 15 publications and 790 citations (Table 2).

**Table 1:** General information of top-five countries and institutions and brand-affiliated institutions with most publications.

Rank	Country/Institution	Publications	Citations	Centrality	Average citations
	Country				
1	United States	475	14,371	0.41	30.25
2	China	236	3,842	0.43	16.28
3	United Kingdom	172	2,865	0.31	16.66
4	Australia	146	2,651	0.10	18.16
5	Canada	138	2,335	0.08	16.92
	Institution				
1	University of Calgary	64	1,838	0.08	28.7
2	Ningbo University	51	607	0.02	11.9
3	Hong Kong Polytechnic University	45	1,193	0.10	26.5
4	University of Massachusetts	39	1,135	0.08	29.1
5	Harvard University	38	3,227	0.10	84.9
	Brand-affiliated Institution				
1	Li-Ning	22	277	0.03	12.6
2	Adidas	15	224	0.02	14.9
3	Decathlon	12	301	0.03	25.1
4	Salomon	11	172	0.01	15.6
5	Nike	10	801	0.05	80.1

**Table 2:** Top-five funding sources with the most publications on running shoes research.

Rank	Funding sources	Region	Number of publications	Total citation count	Average citation count
1	National Natural Science Foundation of China	China	85	1,556	18.3
2	K C Wong Magna Fund in Ningbo University	China	33	570	17.3
3	Coordenação de Aperfeiçoamento de Pessoal de Nível Superior	Brazil	19	288	15.2
3	Natural Sciences and Engineering Research Council of Canada	Canada	19	325	17.1
4	European Union	European Union	16	186	11.6
4	UK Research and Innovation	United Kingdom	16	158	9.9
4	United States Department of Health and Human Services	United States	16	809	50.6
5	National Institutes of Health	United States	15	790	52.7

### 3.3 Analysis of institutions and authors

**Figure 3:**
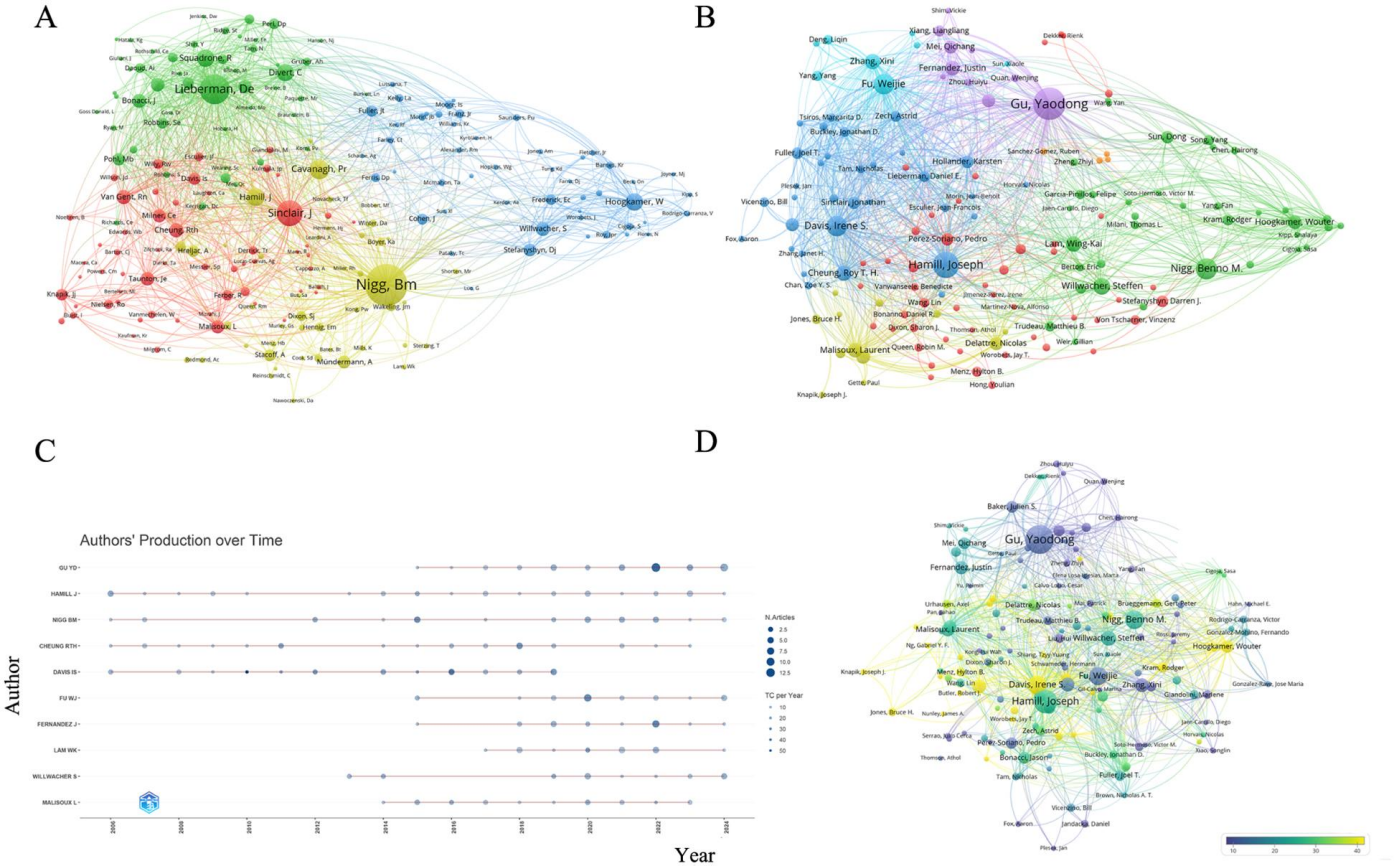
Visualization of author networks. **(A)** The co-citation analysis of authors: the size of the nodes represents the number of citations. **(B)** The bibliographic coupling analysis of authors: the size of the nodes represents the number of articles. **(C)** Total publication output of the author over time. **(D)** The citation analysis of authors: the size of the nodes represents the number of documents. The color represents the average year.

For research institutions, the University of Calgary ranked the first with 64 published articles, followed by Ningbo University (51 articles), the Hong Kong Polytechnic University (45 articles), the University of Massachusetts (39 articles), and Harvard University (38 articles). Among high-publishing authors, Gu YD led with 44 papers, followed by Hamill J and Nigg BM with 25 and 23 articles, respectively. Both Davis IS. and Fu WJ have published 22 articles, while Willwacher S contributed 17 articles. Notably, Gu YD was affiliated with the second-ranked institution, Ningbo University, Hamill J comes from the fourth-ranked University of Massachusetts, and Davis IS associated with the fifth-ranked Harvard University (see Tables 1, 3). Despite there was smaller publication volume of 38 articles for Harvard University, it received an impressive citation count of 3,227, indicating its significant research impact. The average citation rate per paper was 84.9, with a centrality of 0.10. Similarly, although Davis IS published only 22 papers, his average citation rate reached 125.46, further highlighting the importance of her research contributions (see Table 3). The institutional collaboration analysis (see Figure 2D) revealed a substantial partnership between the University of Calgary and the University of Massachusetts, indicating frequent collaborative efforts between Canada and the United States. Furthermore, the strong connection between Ningbo University and the Hong Kong Polytechnic University was identified. Despite Ningbo University's relatively recent emergence, its publication volume was impressive, which reflected its active engagement in running footwear research. Figure 3C illustrates the trend of authors' publication volumes over time, demonstrating that Gu YD and Fu WJ have significantly increased their outputs in the past decade, while Hamill J, Nigg BM, and Davis IS continued to play leading roles in advancing running footwear research. Among brand-affiliated institutions (Table 1), Li-Ning Company led significantly in publication volume (a total of 22 articles), followed by Adidas (15 articles), Decathlon (12 articles), and Salomon (11 articles). Although Nike ranked fifth with 10 published articles, it exceled in both centrality (0.05) and average citation counts (80.1), implying its considerable influence in the field of footwear research.

**Table 3:** General information of top-five authors with most publications.

Rank	Author	Country/Institution	Publications	Citations	Centrality	HI	Average citations
1	Gu YD	China	44	585	0.00	14	13.30
		Ningbo University					
2	Hamill J	United States	25	872	0.02	17	34.88
		University of Massachusetts					
3	Nigg BM.	Canada/Switzerland	23	698	0.00	17	30.35
		University of Calgary					
4	Davis IS	United States	22	2,760	0.01	22	125.46
		Harvard University					
4	Fu WJ	China Shanghai	22	342	0.00	11	15.55
		University of Sport					
5	Willwacher S	Germany	17	439	0.00	10	25.82
		University of Freiburg					

### 3.4 Analysis of journals

Table 4 presents the publication volume and academic impact indicators of the top five journals in the field of running footwear research. *Gait & Posture* ranked the first with 94 published articles, followed by *Journal of Biomechanics* with 65 articles, reflecting the activity and research influence of these journals in this field. All five of the most publishing authors have published in these journals; Specifically, Benno M. Nigg has contributed 26 articles, 13 of which were explicitly running shoe-related modifications. In impact factor, *Medicine & Science in Sports & Exercise* had the highest at 4.1, demonstrating its outstanding reputation in sports science. Although *Gait & Posture* had a lower impact factor of 2.2, it led in publication volume and h-index, suggesting its broader research topics and audience. Citation counts were also critical for assessing academic influence, with *Medicine & Science in Sports & Exercise* showed the highest citation count (4,057) and average citation rate (115.9) for high academic value. Highly-cited investigators such as Joseph Hamill, Benno M. Nigg, and Irene S. Davis published frequently in this journal.

**Table 4:** General information of top-five journals with most publications.

Rank	Journal	Publications	IF	Citations	HI	Average citations
1	*Gait & Posture*	97	2.2	2,828	31	29.15
2	*Journal of Biomechanics*	68	2.4	3,410	27	50.14
3	*Journal of Sports Science*	64	2.3	1,132	20	17.69
4	*Sports Biomechanics*	59	2.0	519	14	8.79
5	*Medicine & Science in Sports & Exercise*	36	4.1	4,147	27	115.19
6	*Plos One*	35	2.9	626	16	17.88

HI, hirsch index; IF, impact factor.

### 3.5 Analysis of keywords

#### 3.5.1 Keyword co-occurrence analysis

**Figure 4:**
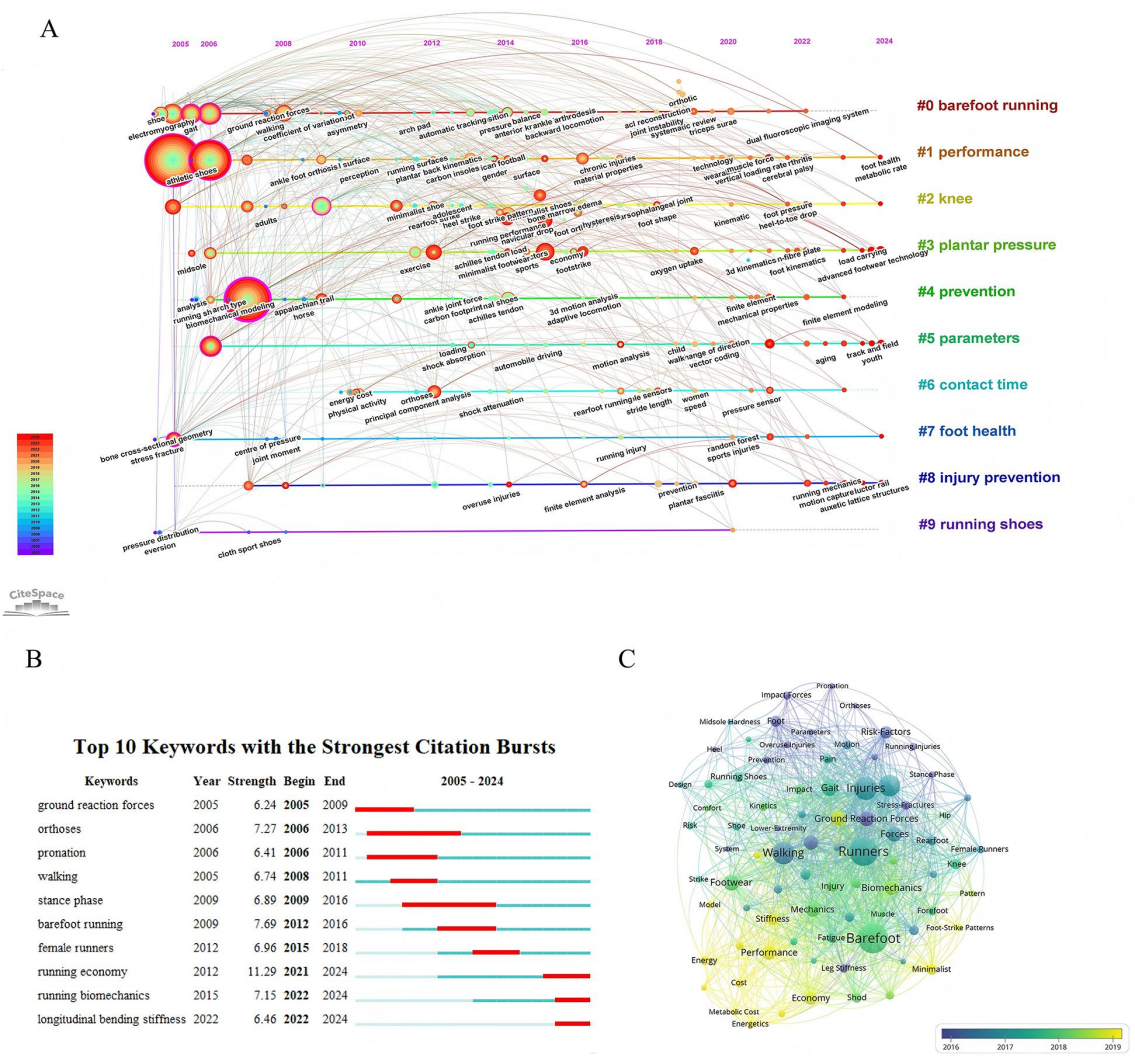
Visualization of keywords and references. **(A)** Timeline visualization with keyword clustering on running shoes. **(B)** Visualization of the top-10 keywords with the most citation bursts: The strength indicates the citation burst intensity; the thin blue-dashed line represents the periods of no burst or the end of a burst, while the thick red-dashed line denotes the start and end of the burst and its duration. **(C)** The co-occurrence view of the keywords: different colors are assigned to keywords based on their average occurrence time. Blue keywords appeared earlier than yellow keywords.

Keyword analysis revealed the research hotspots in the field of running shoes. Figure 4C shows that “barefoot” (295), “runners” (270), “injuries” (198), and “kinematics” (180) were the most frequently occurring core keywords, which indicated that researchers were exploring the biomechanical effects of barefoot running and minimalist shoes, as well as their impacts on athletic performance and injury risk. High-frequency keywords included “injury,” “loading rate,” “comfort,” “cushioning,” and “plantar pressure” were linked to joint loading and foot comfort, which further support the running shoe design that has increasingly emphasized the multifaceted considerations of athletic performance and injury prevention. Additionally, the keywords included “electromyography”, “oxygen consumption” and “running economy” reflected the interdisciplinary research trends among physiology, engineering, and biomechanics, which indicated a growing focus on energy metabolism and muscle function. Further examination of the temporal evolution of research themes (Figures 4A,C) revealed that the development trend of core keywords accelerated between 2016 and 2019, particularly in the areas related to barefoot running and minimalist shoes. This shift would be closely associated with the upcoming public health awareness and the ongoing innovation in running shoe industry.

#### 3.5.2 Keywords bursts analysis in running shoes

The analysis of burst keywords effectively identified current research hotspots, with ten high-burst keywords extracted using CiteSpace (Figure 4B). Temporal characteristics revealed how specific topics have rapidly captured public attention within the running shoe field. Notably, the earliest emerging burst keyword was “ground reaction force.” However, it is important to acknowledge that biomechanists began the investigation on this topic since the late 20th century (11), indicating that the observed trend may be influenced beyond our studied time period. After 2009, interest in this keyword declined in running footwear research. The burst strength of the keywords reflected their relative research attention, with “running economy” achieving a burst strength of 11.29 in 2021, which was significantly higher than that of other keywords and highlighting its status as a core research focus (29). Keywords such as “walking” and “female runners” exhibited persistence to signify their persistent relevance in the field (30, 31). Additionally, emerging terms including “running biomechanics” and “longitudinal bending stiffness” illustrated the innovative studies researchers were currently under explored (32, 33).

#### 3.5.3 Cluster analysis in running shoes

Keyword cluster analysis revealed various research topics within the field of running footwear. By integrating the core terms from each cluster, the overall research framework can be summarized into four interrelated sub-topics: (a) Foundational biomechanics of running, (b) Performance optimization, (c) Injury and damage prevention, and (d) Design parameters of footwear.

##### 3.5.3.1 Fundamental biomechanics of running

The existing literature can be parsed into three dominant clusters. i. Barefoot running (Cluster 0) concentrated on impact attenuation and landing strategy: the keywords “barefoot” (*n* = 307) and “runners” (*n* = 274), which established the barefoot condition as the reference model against which shoe cushioning and propulsion structures were evaluated (32). The additional prominence of “force” (*n* = 109) underscored the key role of ground reaction forces in this field (34). ii. Plantar pressure (Cluster 3) linked the pressure distribution to injury risks, which had the co-occurrence of keyword “plantar pressure” (*n* = 92) and “risk factors” (*n* = 85) pointing to a strong association between forefoot pressure hotspots and overuse injuries such as metatarsal stress fractures and metatarsalgia (35). iii. Contact time/Gait analysis (Cluster 6) reflected the prevailing experimental paradigm. The keywords “gait analysis” (*n* = 37), “distance runners” (*n* = 34) and “treadmill” (*n* = 23) revealed that most investigations were carried out on constant-speed treadmills, with synchronized acquisition of force-plate, motion-capture and metabolic data under controlled testing conditions (29, 36).

##### 3.5.3.2 Performance optimization

Running economy is defined as the oxygen cost per unit body mass and per unit distance at a constant speed (ml kg⁻¹ km⁻¹) and typically measured with open-circuit spirometry, which served as the physiological benchmark for footwear evaluation (31). From a biomechanical perspective, the same construct has been inferred from external mechanical variables such as ground-reaction forces, joint work and spatiotemporal gait parameters (37). Bibliometric analysis resolved two complementary knowledge clusters. i. The “Performance” (Cluster 1) was dominated by the keywords “performance” (*n* = 98), “economy” (*n* = 81) and “running economy” (*n* = 59), which suggested that improvements in running economy have become a primary criterion by which both academia and industry running shoes (29). ii. the “Parameters” (Cluster 5) was characterized by “ground reaction forces” (*n* = 119), “biomechanics” (*n* = 102) and “impact forces” (41), which reflected that researchers have investigated the mechanisms of running economy through quantified external dynamics (1).

##### 3.5.3.3 Injury and damage prevention

The incidence of running-related injuries ranges from 20%–70%, with the knee, tibia, and Achilles tendon being the most common parts of injury (3, 12, 13). Therefore, running injuries has been a significant research focus within the field of sports biomechanics. The included articles can be analyzed through four major clusters: “Knee” (Cluster 2), “Prevention” (Cluster 4), “Foot health” (Cluster 7), and “Injury prevention” (Cluster 8). i. The keywords in Cluster 2 include “injury” (*n* = 277), “kinematics” (*n* = 187), and “footwear” (*n* = 115). Research indicates that the design and type of running shoes significantly influence the kinematic characteristics of the knee and the associated injury risk. Consequently, the appropriate selection of running shoes and adjustments to running posture become key measures for reducing knee injury potential (13, 38). ii. Cluster 4 included keywords included “overuse injury” (*n* = 41), “lower extremity” (*n* = 37), and “pressure” (*n* = 37). Overuse injuries represented a significant aspect of running-related injuries in the lower extremities. Research indicates that the distribution of pressure and movement patterns during running are critical for maintaining lower limb health. Implementing a well-structured training plan and appropriate shoe selection may reduce the risk of overuse injuries (39, 40). iii. Cluster 7 contained keywords included “reliability” (*n* = 64), “impact” (*n* = 59), and “motion” (*n* = 45). This highlights the close relationship between foot health and shoe design, particularly concerning impact force management and the influence on movement patterns (41). The cushioning design of running shoes aimed to attenuate the impact on foot, and the effectiveness of this design depends on both the reliability of the shoes and the individual movement characteristics of the runner (42). iv., the keywords in Cluster 8 included “model” (*n* = 35), “injury prevention” (*n* = 28), and “behavior” (*n* = 16). The research on injury prevention emphasized the importance of developing effective models to predict and reduce running injuries (43). By analyzing level and patterns of runners, footwear features, and exercise biomechanics parameters, potential risk factors can be identified for optimizing appropriate prevention strategies (44, 45).

##### 3.5.3.4 Design parameters of footwear

Running shoes (Cluster 9) featured the keywords included “running shoes” (*n* = 103), “risk” (*n* = 33), “Achilles tendon” (*n* = 19), and “shape” (*n* = 10). These keywords highlighted the significant impact of shoe design on athletic performance and injury risks (46). i. The design of running shoes is directly related to running performance and comfort. Modern running shoes typically include features such as cushioning, support, and stability, which aimed to enhance running economy and reduce the risk of injuries (7). ii. Regarding the keyword “risk”, previous studies indicate that improper shoe design could increase injury risk, particularly concerning the Achilles tendon and knee joint (40). iii. Surrounding the keyword “Achilles tendon”, it is a critical area subjected to substantial forces during running, and the design of running shoes plays a significant role in managing its load and strain (47).

### 3.6 Analysis of references

By summarizing the top-ten highly cited articles in this study (Table 5), Lieberman et al. (32) undoubtedly hold a central position due to their highest citation rate and publication in a prestigious journal (Nature). Their concept of “barefoot running” has directly influenced subsequent footwear research directions, as evidenced by the volume of annual publications related to running shoes nearly tripled in 2012 following the release of their paper (Figure 2A). The work of Lieberman DE also holded significant importance in citation metrics (Figure 3A), while “barefoot” emerged as the most frequently used keyword in footwear research (Figure 4C). Among the top-ten cited publications, six of them focused on “barefoot running,” that explored the effect of barefoot running on both runner performance and injury risk by using biomechanical, physiological, and cross-sectional analyses. Additionally, a review by Moore et al. (37) suggested that variations in running techniques may help reduce injuries and enhance performance, although the design of shoes appears to play a more critical role. The study done by Hoogkamer et al. (29) on the Nike Vaporfly 4% shoe induced significant attention and the transition of running shoe research into a phase of rapid growth. Researchers have then began to investigate how different shoe structures, along with carbon plate design and integrations, could improve running economy and performance.

**Table 5:** Top-ten high-cited articles in running shoes.

Rank	Article title/Study design	First author	Instruments/Methods	Variants	Journal/Year	Average citations
1	Foot strike patterns and collision forces in habitually barefoot vs. shod runners/Lab-based biomechanics study	Lieberman DE	Motion capture; Force plate	Peak vertical force; Loading rate	*Nature*/2010	62.3
2	Foot strike and injury rates in endurance runners: a retrospective study/Retrospective Cohort Study	Daoud AI	High speed camera; Running Injury Severity Score	Injury rates	*Medicine & Science in Sports & Exercise*/2012	25.9
3	A kinematics and kinetic comparison of overground and treadmill running/Lab-based biomechanics study	Riley PO	Motion capture; Force plate	Gait parameter; Lower limb angle and moment; Ground reaction force	*Medicine & Science in Sports & Exercise/*2008	18.9
4	Biomechanical and physiological comparison of barefoot and two shod conditions in experienced barefoot runners/Lab-based biomechanics study	Squadrone R	High speed camera; Plantar pressure treadmill; Portable metabolimeter	Gait parameter; Running economy	*Journal of Sports Medicine and Physical Fitness*/2009	19.3
5	A comparison of the energetic cost of running in marathon racing shoes/Lab-based biomechanics study	Hoogkamer W	Motion capture; Force plate; expired-gas analysis system	Metabolic power; Vertical ground reaction force	*Sports Medicine*/2018	36.1
6	Effects of footwear and strike type on running economy/Lab-based biomechanics study	Perl DP	Motion capture; Force plate; Open-flow respirometry system	Running economy; Lower limb angle and moment; Ground reaction force	*Medicine & Science in Sports & Exercise*/2012	16.7
7	Is there an economical running technique? A review of modifiable biomechanical factors affecting running economy/Review	Moore IS	Review	Running Economy; Cadence; Leg Stiffness; Joint kinematics and kinetics	*Sports Medicine*/2016	20.7
8	Mechanical comparison of barefoot and shod running/Lab-based biomechanics study	Divert C	3D forces treadmill; Bilateral bipolar surface-EMG	Ground reaction force; Mean amplitude	*International Journal of Sports*/2005	27.6
9	Barefoot running: biomechanics and implications for running injuries/Review	Altman AR	Review	Running Economy; Joint kinematics and kinetics; Ground reaction force; Arch strain; Plantar-flexor impulse	*Current Sports Medicine Reports*/2012	14.6
10	Forefoot strikers exhibit lower running-induced knee loading than rearfoot strikers/Lab-based biomechanics study	Kulmala JP	Motion capture; Force plate; Hand-held dynamometer	Gait parameter; Lower limb angle and moment; Ground reaction force	*Medicine & Science in Sports & Exercise*/2013	14.9

Regarding the specific research settings for running studies, Riley et al. (36) analyzed the biomechanical differences between treadmill and overground running, and demonstrated that treadmill testing could serve as an effective research setting. This finding offers new possibilities for instrumented treadmill utilizing motion capture and three-dimensional force plate instruments in quantitative assessments of running. Methodologically, the ten most-cited articles converge on a remarkably similar experimental protocol and toolbox. Eight of the empirical articles combined high-speed 3-D motion capture (120–500 Hz) with either floor-embedded force platforms or dual-belt instrumented treadmills sampling at 1–5 kHz to quantify foot-ground interactions and joint kinetics; four articles further incorporated open-flow or portable metabolic carts to estimate energetic costs as oxygen consumption or running economy (oxygen consumption/speed); and two articles augmented these setups with surface electromyography to resolve plantar-flexor activation patterns. Consequently, the most commonly used outcome variables were peak vertical ground reaction force and loading rate, running economy or metabolic power, lower-limb joint angles and moments, and spring-like strains of the medial arch and Achilles tendon. Among these 90 extracted keywords from the ten highly cited papers, only a few keywords were reoccurred with “forefoot strike” appeared three times, whereas “ground reaction forces”, “foot strike patterns” and “leg stiffness” appeared twice each. Foot-strike modality—forefoot vs. rearfoot—thus emerged as the primary focus. Mechanical determinants (ground reaction forces, limb stiffness), energetic metrics (metabolic cost) and treadmill-based experimental designs constituted the secondary but recurrent themes. Notably, among these top-ten highly cited articles, seven of them were based on laboratory biomechanical research, which indicated that the primary research design was currently carried out in biomechanical laboratory.

## 4 Discussion

### 4.1 General research profile

This study aimed to conduct a comprehensive bibliometric review on running footwear studies in the Web of Science Core Collection database from the Web of Science published from 2005–2024. To explore the research and direction status, we utilized Biblioshiny, VOSviewer, and CiteSpace to conduct a visual analysis of the publication characteristics and research hotspots within the field of running footwear (Figure 1). Over the 20-year period, the annual growth rate in this field was 13.5%, with an average citation rate of approximately 24 times per publication. Notably, the publication volume in the running footwear showed two rapid growth periods in 2011 and 2018 respectively (Figure 2A), and culminated in a peak in 2022 with 152 publications. These would be associated closely with the widespread interest in “*minimalist shoes*” and “*super shoes*” concept (3, 29).

The current results indicated that the United States had the leading position in both publication quantity (30.1%) and citation frequency, with its total citation count larger than the combined total of the other four leading countries (China, the United Kingdom, Australia and Canada, see Table 1). It would be related to the gross domestic product output and supportive policy of athletic development. The previous research highlighted a strong positive correlation between the total number of publications and gross domestic product (48), with the United States and China projected to rank at the forefront of global gross domestic product in 2024 (https://data.worldbank.org.cn/). In 2025, the revenue of the sports market is expected to reach $52.77 billion (https://www.statista.com). The great sports market potential, along with supportive policies from the United States, has attracted a large number of great athletes migrated from around the world to register the National Collegiate Athletic Association of the United States. These athletes and events have, to some extent, generated greater research demands and collaborations with numerous research institutions across the United States (Table 1). Notably, early influential articles on running shoes primarily originated from the United States (49, 50), including one notable article from Nike (51). Although China has lagged behind the United States in both publication and citation totals, China exhibited impressive centrality metrics to indicate its engagement and potential in international collaboration.

Further analysis of institution revealed that the top-five publication volume institutions were one from Canada and two each from China and the United States, which further highlighting the impact of both China and the United States in the running footwear research. The University of Calgary had the highest publication volume (64 articles), followed by Ningbo University (51 articles). Despite similar publication volumes, there exists a significant differences in citation rates, which is consistent with national citation characteristics (Table 1). Notably, Harvard University had only low ranking in publication volume, but achieved the highest citation count (3,227 citations) and exhibited the highest centrality index (0.1) in the running footwear research. Among shoe brands, Li-Ning had the publication volume (22 articles), followed by Adidas (15 articles) and Decathlon (12 articles). This demonstrated Li-Ning has been active engagement in footwear research in the past two decades. Interestingly Nike had fewer publications (10 articles) but holded the highest citation count (801 citations), which implying its authoritative position in shoe industry. Another explanation could be that Nike has the strong collaboration and contribution (e.g., sponsorship for Footwear Biomechanics Symposium, postgraduate scholarship and project funding) with the high-ranking institution to promote the footwear research in the community.

For the author analysis (see Table 3), Gu YD from Ningbo University ranked the first in publication volume (44 articles), who focused on research direction with the keywords included “*Biomechanics*”, “*Gait*”, “*Footwear*”, and “*Lower Limb*”. Conversely, Davis IS from Harvard University occupied a central role in the citation network with a citation count reaching 2,760, and focused on “*lower extremity biomechanics*”*, “gait*”*, and* “*injury*”. Our literature analysis indicates that Lieberman DE's 2010 article on “barefoot running” published in *Nature*, occupied a central position in running footwear research (36.1 citations per paper). Another significant article published by Hoogkamer et al. in “*Sports Medicine*” on “*super shoes*” was the second-highest citation rate. This further supports that both articles hold the key positions in the current citation network of running footwear research. The current funding analysis revealed (Table 2) that the National Natural Science Fund of China supported up to 85 papers in shoe research and the total of this China's funding was larger than the total funding amount contributed from the other four funding sources, which highlighted the China has provided substantial support for this running footwear research area. Publication analysis revealed that the “*Gait & Posture*” was the most published journals (97 articles), while “*Medicine & Science in Sports & Exercise*” was identified as the most influential journal (4,147 citations).

### 4.2 Research hotspots and trends

The current study employed comprehensive keyword analysis and bibliometric analysis to identify research hotspots and attempt to predict cutting-edge research in the field of running footwear. Research themes within the bibliometric field can be reflected through cluster analysis of keywords, while the evolution of research hotspots can be revealed through the timeline of clusters and burst keywords (22). Based on Citespace, the current study identified four key themes based on prominent research directions (Fundamental biomechanics of running, performance optimization, injury and damage prevention, and design parameters of footwear). Additionally, the top 10 burst keywords are listed as “*running economy*” (57 occurrences), “*barefoot running*” (307 occurrences), “*orthoses*” (18 occurrences), “*running biomechanics*” (20 occurrences), “*female runners*” (40 occurrences), “*stance phase*” (26 occurrences), “*walking*” (198 occurrences), “*longitudinal bending stiffness*” (27 occurrences), “*pronation*” (18 occurrences), and “*ground reaction forces*” (86 occurrences). The future studies would extend from this direction with the use of advanced measurement technologies (e.g., IMU, markerless system, blood oxygen). Wearable technologies would allow the running footwear evaluation from laboratory to outdoor running conditions.

In running, joint dynamics, spatiotemporal parameters, and muscle activity collectively form a key contributing factor triangle for understanding injury mechanisms and performance (49). Researchers have developed musculoskeletal models to simulate the interactions between muscles and bones, dynamic models (which simulate temporal-mechanical behavior), and finite element models to assess stress and strain in the musculoskeletal system under load (52–55). These represent the most prevalent modeling approaches among the included running biomechanics studies. Our data (Table 5) indicate that over 70% of the top-10 most-cited articles employed musculoskeletal modeling, which captured the data from force plates and motion capture systems. Among the leading authors, all of them have conducted studies on footwear design using musculoskeletal models, with Nigg BM being among the earliest and most prominent researchers to link muscle-bone modeling with shoe innovation and development (2). Musculoskeletal modeling have been focused on the joint dynamics included the parameters included joint angles, velocities, forces, moments, and work during running, typically calculated using Visual 3D (52). Unlike this inverse dynamics approach that commonly applied with Visual3D, dynamic models such as finite element model emphasize the interactions within multibody systems (muscles, joints, and bones) and their nonlinear biomechanical behavior using both forward and inverse dynamics. Softwares such as OpenSim and AnyBody can support both forward and inverse dynamics calculations (56, 57). Finite element models concentrate on the mechanical behavior of the lower limbs during running, using numerical analysis to evaluate stress and strain under various loads, with ANSYS and ABAQUS as frequently utilized software (58, 59). Among the top-five publishing authors in the current analysis, only Gu YD has employed finite element analysis approach for shoe design research (Table 3). In the sub-topics of fundamental biomechanics of running (Figure 4), Cluster 0 “*barefoot running*” primarily focused on the keywords of “*foot strike patterns*”, “*stress fractures*”, and “*force*” and then gradually shifting towards “*minimalist shoes*” and “*stiffness*”. This indicates that earlier studies mainly concentrated on confirmation of the benefits of barefoot running (60) and its mechanism to mitigate the risks of stress fractures and force through barefoot running (43, 60). Recent research has shifted its focus how minimalist shoes can reduce lower limb stiffness during initial contact, potentially due to the shoe construction that can encourage runners to adopt a more natural gait (60). For the Cluster 3 “*plantar pressure*”, the research trend has transitioned from foot pronation analysis to fatigue assessment. Some current studies are suggested to revise the traditional paradigm of “*injury caused by excessive foot pronation*” (13) into placing greater emphasis on the fatigue effects on the foot (61). Furthermore, one research trend on Cluster 6 “c*ontact time”* has shifted from “*energy cost”* and “*shock attenuation”* to “*wearable sensor”* and “*pressure sensor*” (Figure 4A). This indicates that with advancements in wearable and sensor technologies, researchers are increasingly applied these devices for data collection related to contact time (62).

Over the past few decades, researchers have made significant efforts to enhance running economy in laboratory settings through various training and intervention. Running economy is defined as the rate of oxygen consumption at a given sub-maximal running speed and it is the most commonly used physiological indicator for assessing endurance running performance (63). In the biomechanics perspective, the analysis of running economy often focuses on mechanical efficiency. Existing research indicates that greater stride length, specific muscle activation patterns (such as reduced muscle tension and improved coordination), smaller vertical displacement of the pelvis, limited knee joint range of motion, lower horizontal velocity of the pelvis, and shorter contact times or contact time ratios are associated with higher running efficiency and improved race duration (64–66). Within the sub-topic of “*Performance Optimization*”, the research focus has shifted from “*3D kinematics*” to “*muscle force*” and “*metabolic rate*,” which indicated that the analysis of running shoe characteristics by integrating multiple metrics would become a key trend in future studies for comprehensive evaluation. For instance, among the top-10 most cited papers (Table 5), several studies have combined motion capture, force platforms, and gas analysis system (29, 90, 91). By integrating biomechanical variables with metabolic indicators, a more effective explanation of running performance can be achieved (64).

There are no specific keywords related to footwear features under the sub-topic “*Design parameters of footwear*”. However, our analyses revealed that footwear characteristics and modifications such as “*midsole*” appeared in the cluster of “*plantar pressure*” and “*heel-to-toe drop*” appeared in cluster of “*knee*”. The shoe characteristics of interest included shoe weight, comfort, midsole thickness and elasticity (material properties), heel-to-toe drop, cushioning characteristics, and the shape and stiffness of carbon plates (67, 68). Previous research suggested running shoes with smaller shoe mass can generally improve running economy (69). Additionally, running shoes with better perceived comfort can significantly reduce oxygen consumption by about 0.7% (70). Regarding midsole elasticity, the two common midsole materials (EVA and PU) reveal that EVA is relatively lightweight that can provide good comfort and energy feedback but show poor durability (71). The current research indicates that variations in the heel-to-toe drop have no significant effect on spatiotemporal and lower limb kinematics variables during running, but lower heel-to-toe drops may lead to higher vertical loading rates (72). Longitudinal bending stiffness is a hot topic in running shoe research (Figure 4B). Although the large volume of publication on bending stiffiness appears to have surged in 2022, the earliest investigation was published back to 2006 (73). In a review study done on the effects of increased longitudinal bending stiffness on running efficiency, the findings were inconsistent across studies. This discrepancy may be resulted from the position and shape of carbon plates within running shoes that would have influenced on running economy. Therefore, further research is needed to explore the interactions between carbon plate designs and other shoe features such as midsole properties and midsole geometry, as well as how these traits vary with shoe size, body weight, and individual landing patterns (74). However, current studies on longitudinal bending stiffness remain contradictory. Some studies indicate that increased longitudinal bending stiffness can significantly enhance running economy (75), while other studies found no significant effect on the running economy of shoes (5). Notably, Nike Vaporfly 4% has attracted significant attention in academia. This shoe has been showed to play a crucial role in helping the sponsored marathon runner break the 2-hour barrier (29). The footwear studies indicated that participants wearing this carbon plate shoe can enhance running economy by approximately 4%, despite individual differences (9, 29). Our data show that Hoogkamer et al.'s study was the top-five most cited article, and we speculate that the rapid increase in annual publications in 2018 may be linked to the Nike Vaporfly 4% concept. Nigg et al. (76) introduced the “*teetertotter principle*” proposed that the curved/spoon-shape carbon fiber plate and forefoot curvature can shift ground reaction forces forward during propulsion, yielding additional upward thrust at takeoff. This mechanism highlighted the importance of carbon plate geometry and stiffness in enhancing propulsion and running economy (76). Among the several footwear brands (Table 1), Nike did not contribute the largest publication volume, but it is the most influential company. The top-10 most cited papers included the study by Hoogkamar et al. (29), which were sponsored by Nike. The primary research directions of Nike company have shifted the research focus on various types of footwear (e.g., motion control and minimalist shoes) on athletic performance, injury incidence rate (77), and innovative shoe materials on running economy (78). In the field of biomechanics, the top-five footwear companies in publication volume have utilized similar equipment to assess the effects of running shoes on running injuries and performance (29, 46). All of these companies have analyzed running economy through gas metabolism parameters (79–82), although there were variations in specific metrics and protocols, with Nike's earlier study (29). On the other hand, Adidas has employed finite element analysis (83) and machine learning techniques (84) to study footwear characteristics, highlighting its innovative position within the industry. These findings suggest that Nike has initiated testing protocols of running economy with high scientific recognition, while Adidas has demonstrated good potential at structural innovation. Finite element analysis demonstrates significant potential in footwear research, as it can effectively assess the impact and stress of various shoe features on injuries in a virtual environment (45). By integrating traditional biomechanical testing methods, this approach provides a deeper understanding of the stress variations within the lower extremities, and thereby offering a theoretical basis for optimizing running shoe design (54). The finite element analysis was identified as a new research hotspot across several clusters (Figure 4A). For instance, “*finite element analysis*” can be appeared under the cluster of “*injury prevention*”, while “*finite element mechanical properties*” and “*finite element modeling*” can be found under cluster of “*prevention*”. These findings indicate that finite element analysis is frequently utilized to assess the injury prevention in running shoes (54).

Currently, running shoes can be classified into different types included minimalist shoes, motion control shoes, and cushioned running shoes, based on functionality, construction, and design philosophies. Motion control shoes are designed to offer support and stability for overpronated or heavy runners with firmer midsole materials at medial side and higher heel-to-toe drops (Cluster 3 “*midsole*”), while cushioned running shoes are designed to maximize comfort and shock absorption (Cluster 3 “*shock attenuation*”) with thicker midsoles and elastic cushioning materials (1). The goal of minimalist shoes aims to restore the natural movement and foot function whilst minimizing the interference and protection by the shoes (3). These shoes are typically featured with thinner outsoles, lower heel-to-toe drops, lighter shoe mass, and minimal cushioning, allowed for runners accustomed to barefoot running or who are planned to train to adapt a more natural running style (32). The philosophy lies on the keyword “*barefoot running*” with Lieberman group who has conducted several influential studies related to this topic (32, 60, 85). The burst analysis indicates that barefoot running began to gain attraction in 2009 and became a footwear research focus from 2012–2016 (Figure 4B). Within Cluster 2, the burst of the keyword “*minimalist shoe*” occurred approximately between 2010 and 2012 (Figure 4A). With both burst term and timeline analyses, the Lieberman's research group has made significant contribution to advance the research on minimalist shoes. Minimalist shoes may reduce injury risk through biomechanical mechanisms that decrease vertical impact peaks and contact times and these barefoot-like footwear can encourage runners to return to more natural movement patterns, potentially helping to reduce lower limb injury risks (3). Analysis of the top-ten most cited publications reveals that seven of these studies have focused on “*foot strike pattern*” (Table 5). Timeline analysis indicates that since 2010, keywords such as “*foot strike pattern*” (Cluster 2), “*ankle joint force*” (Cluster 4), and “*overuse injuries*” (Cluster 8) have been widely discussed (Figure 4A). These studies provide important theoretical foundations for foot strike patterns and optimization of minimalist shoes.

### 4.3 Challenges in current research and future development directions

In running footwear evaluation and research, several challenges have persisted. To our best knowledge, the burst keywords “*running biomechanics*” and “*ground reaction forces*” have long been considered as the key focus on footwear research. Our results may reflect the reviewed time period chosen for our bibliometric study, rather than a sudden change of research interest. Furthermore, some methodological concerns about near-duplicate keywords across clusters. Under the “*Fundamental biomechanics of running*” theme, for examples, the keyword “*foot strike patterns*” appeared 108 times, whereas the shorter keyword “*foot strike*” occurred 57 times. Similarly, under “*Performance optimization*” theme, “*economy*” (81 times) co-existed with “*running economy*” (59 times). Under “*Injury and damage prevention*” theme, “i*njury prevention*” (28 times) duplicated “prevention” (28 times). Such overlap highlights conceptual ambiguity within the field. We therefore advocate 1. a more explicit keyword-selection strategy, 2. standard experimental designs, and 3. standardize the definition of “injury” with community consensus. Greater semantic precision can improve methodological precise and establish a clearer and more coherent conceptual framework for future studies.

The running biomechanics and gas metabolism are two major methods in running shoe research. Running biomechanics is typically assessed at a fixed speed, as foot strike angle, lower limb positioning and biomechanical parameters vary at different running speeds (34). To measure gas metabolism during running, personalized speed settings are often employed to accurately assess individual runner's metabolic efficiency (63). Such difference in testing speed setting may affect cross comparisons between the two fields of research. Regarding data analysis, current research often relies too heavily on comparisons of the group means, but overlook individual differences. This statistical approach may lead to weak correlations or high variance to derive clear causal relationships (86). Existing literature indicates that the understanding of the biomechanical mechanisms remains insufficient, particularly as most studies utilizing biomechanical parameters to explain running economy and injury risks that is appeared to lack adequate evidence (87, 88). A study addressed running injuries also confirmed a lack of empirical evidence on this relationship (12). Regarding footwear-injury research, the explanation based on a single biomechanical indicator is limited, and several long-term studies are frequently interrupted by various reasons. Biomechanical characteristics, injury risks and footwear preference can vary with running proficiency (66). Novices has less stable gait and injury-prone, and therefore tend to choose soft, highly cushioned shoes for impact attenuation (39). Recreational runners are susceptible to technique breakdown under fatigue, and therefore seek footwear with good cushioning with energy return and/or medial and lateral support (89). Elites possess stable mechanics and high running economy, and therefore favour lightweight, performance-oriented shoes (46). Therefore, future research should adopt multimodal data collection methods to thoroughly investigate the causal relationships among biomechanical parameters, injuries, and performance. Additionally, future studies should consider different running skill levels, gender, running style, and injury history. Utilizing cross-sectional study designs can enhance the unity of the findings for more specific keyword policy, experiment registration, and the consensus of injury definition.

There are some limitations when interpreting our results. First, we conducted the literature search in a single database, which may have resulted in the omission of relevant articles that are not retrieved from the single database. Second, only English-language journal articles have included in our analysis, non-English articles, conference proceedings or PhD theses may introduce selection bias and reduce accuracy. Notably, significant contributions from China, Brazil and other non-English speaking countries may be under presented. Third, the searching period from 2005–2024 may exclude the key articles published before 2005, which could have overlooked some early research in the field of running shoe studies. Although these footwear variables have been addressed and discussed in subsequent studies, these may limit the comprehensiveness of the research. Fourth, the field of running footwear research is rapidly evolved, with new findings emerging, therefore additional studies in this field may not have been included. The findings from this study can provide a useful overview of the current status and trends, offering valuable directions for future research.

## 5 Conclusion

This study presented the first bibliometric analysis of running footwear research to identify the main research hotspots and future trends over the past 20 years. The annual publications on running footwear have steadily increased. Current research has focused on the biomechanical and physiological indicators to investigate related injury risks and running performance during shod running, with particular attention to the effects of minimalist shoes. Future research should consider how running shoe features would match with individual differences such as foot strike patterns and skill levels. By incorporating running biomechanics and gas metabolism, a comprehensive evaluation can be established to assess the effect and underlying mechanism of footwear features on running. Additionally, simulation analyses can be used to study internal stress changes within the musculoskeletal system. Moreover, employing deep learning methods combined with advanced measurement technologies such as inertial measurement units, markerless systems, and blood oxygen measurement can allow for continued evaluation of running shoes in outdoor running conditions.
